# Reviewed article: Rodrigues EN, Krieck LK, Barbian MH, Schuler-Faccini L, Iser BPM. Prevalence and spatial distribution of congenital heart defects: descriptive study, Santa Catarina, 2011-2019. Epidemiol Serv Saude. 2026:35;e20240212

**DOI:** 10.1590/S2237-96222026v35e20240212.a

**Published:** 2026-04-17

**Authors:** Tania Vignuda de Souza

**Affiliations:** 1 Universidade Federal do Rio de Janeiro, Rio de Janeiro, RJ, Brazil

## Peer review A

## Questionnaire

Does the manuscript contain new and significant information to justify publication? Yes

Does the Abstract (Summary) clearly and accurately describe the content of the article? No

Is the problem significant and concisely stated? Yes

Are the methods described comprehensively? No

Are the interpretations and conclusions justified by the results? Yes

Is adequate reference made to other work in the field? No

## Manuscript Structure

Length of article is: Too short

Number of tables is: Adequate

Number of figures is: Adequate

Please state any conflict(s) of interest that you have in relation to the review of this paper. None

Interest: Good

Quality: Average

Originality: Average

Overall: Average

Recommendation: Minor Revision

## Comments

Parabéns pelo estudo, no entanto, devem ser revistas as seguintes considerações:

No resumo, especificamente na conclusão, os autores afirmam que as cardiopatias possuem uma prevalência intermediária em comparação com a média nacional, no entanto, não foi este o objetivo do estudo.

Na página 3, no último parágrafo antes das fontes de dados, sugiro que na frase “O baixo peso foi detectado para criança com menos de 2500 gramas, sendo extremos quando < 1500 gramas ou ≥ 4000 g” para “O baixo peso foi detectado para criança com menos de 2500 gramas, sendo também considerados os extremos quando < 1500 gramas ou ≥ 4000 g”.

Controle do viés, na página 3, quantos registros foram excluídos do total, referente ao intervalo de 2011 e 2029? Como foram identificados os dados duplicados? A duplicidade se refere aos dados do recém-nascido ou da mãe? Deixar mais claro.

Rever a sigla CID no plural. Receio que não existe CIDs, ainda seria as CID, pois está se referindo a classificação internacional de doenças.

Corrigir o nome da cidade “Santa Amaro da Imperatriz” na página 4.

Rever a ordem das referências, por exemplo, a referência n. 30, na página 5.

Algumas referências poderiam ser atualizadas.

